# Beyond individual skill: How school innovation climate amplifies the pathway from Generative AI Adoption to deep pedagogical integration

**DOI:** 10.3389/fpsyg.2025.1701051

**Published:** 2026-01-21

**Authors:** Tongqiang Dong, Yong Kong, Ronglong Chen, Ziyi Yang

**Affiliations:** School of Communication, Qufu Normal University, Rizhao, China

**Keywords:** generative AI, deep integration, teacher digital literacy, teacher leadership, school innovation climate, multilevel analysis, moderated mediation

## Abstract

**Introduction:**

Highlighting the critical role of organizational context in Generative AI (GAI) integration, this study proposes a multilevel model to explore the interplay between individual teacher development and school-level factors. Specifically, we examine how teacher digital literacy and pedagogical leadership mediate the link between GAI adoption and deep integration, alongside the moderating role of school innovation climate.

**Methods:**

Data from 512 teachers across 45 schools in China were analyzed using multilevel structural equation modeling (MSEM) and hierarchical linear modeling (HLM). This design allowed for the simultaneous assessment of sequential mediation pathways at the individual level and cross-level interactions involving organizational climate.

**Results:**

Results confirmed a sequential mediation: GAI adoption enhances digital literacy, which boosts pedagogical leadership, ultimately driving deep integration. Crucially, school innovation climate functioned as a significant cross-level moderator (*γ* = 0.21, *p* < 0.01); the positive impact of pedagogical leadership on integration was markedly stronger in supportive environments.

**Discussion:**

These findings demonstrate that GAI’s transformative potential relies on a synergy between individual competencies and a supportive organizational environment. The study suggests that to maximize technological efficacy, policymakers and leaders must prioritize cultivating innovation-supportive school climates alongside individual training.

## 1 Introduction

The advent of Generative Artificial Intelligence (GAI) has initiated a profound transformation in society, particularly within education (Zawacki-Richter et al., 2019; Baidoo-Anu and Ansah, 2023; Ryzhova et al., 2023; Williamson et al., 2020). Tools such as ChatGPT and ERNIE Bot offer significant potential to automate administrative tasks, generate tailored learning materials, and support personalized student pathways (Farrokhnia et al., 2023). This technological advance has sparked considerable enthusiasm and swift adoption among educators eager to leverage its efficiencies, with many early reports highlighting its potential to revolutionize educational practices (Shao, 2023). Consequently, emerging literature is examining the factors driving GAI adoption, focusing primarily on individual aspects such as self-efficacy, technology acceptance, and perceived usefulness (Márquez et al., 2023; Wu and Yu, 2023).

A significant gap remains between the initial adoption of GAI tools and their comprehensive pedagogical integration (Timotheou et al., 2023; Yan et al., 2024a). While many educators utilize GAI for efficiency, such as in lesson planning or administrative tasks, fewer employ it to fundamentally redesign learning tasks and create innovative educational opportunities (Alasadi and Baiz, 2023; Tang, 2024; Abulibdeh et al., 2025). This gap indicates that transitioning from tool usage to pedagogical transformation is neither straightforward nor automatic, but rather a complex process hindered by individual and systemic barriers (Mustafa et al., 2024). Current research, often guided by models like TAM or TPACK, fails to fully explain this phenomenon. These models, though useful, tend to decontextualize teacher practice (Chen et al., 2023), neglecting the significant impact of the organizational context (Fullan, 2016; Van Braak et al., 2004). As Tondeur et al. (2015) showed, school-level factors can account for up to 40% of the variance in technology integration, highlighting the limitations of focusing solely on individual factors.

To advance “beyond implementation” toward meaningful change (Fullan, 2016), this study employs an ecological systems perspective (Geesa et al., 2019). This framework suggests that educational change results from the dynamic interplay between individual agency (microsystem) and the organizational environment (mesosystem and exosystem). We propose a multilevel model to explore the micro-mechanisms of this interaction. At the individual level, we theorize a developmental pathway where initial GAI adoption enhances teachers’ digital literacy, empowering them to demonstrate pedagogical leadership, crucial for deep integration. This forms a sequential mediation chain. Additionally, we examine how organizational context influences this individual pathway. We argue that a school’s innovation climate—shared perceptions of institutional support for risk-taking and new ideas (Damanpour, 1991; Van Wijk et al., 2018)—serves as a critical cross-level amplifier. A supportive climate can enhance an innovative teacher’s efforts, while a restrictive one may hinder them, regardless of individual skill. To operationalize this ecological interaction, we conceptualize teachers’ professional development (adoption, literacy, leadership) as the microsystem process, while the school’s innovation climate represents a critical feature of the mesosystem.

Our model thus aims to empirically test how these systems dynamically interact, with individual pathways being either enabled or constrained by the surrounding organizational context. Specifically, we define the individual teacher’s attributes and developmental process (GAI adoption, digital literacy, pedagogical leadership, and deep integration) as Level-1 factors. We conceptualize the school’s organizational characteristics, represented by the innovation climate, as a Level-2 factor that influences these individual-level processes.

Therefore, this study aims to clarify the mechanisms of deep GAI integration through a multilevel lens by addressing two primary research questions:

RQ1: To what extent do teacher digital literacy and pedagogical leadership sequentially mediate the relationship between initial GAI adoption and its deep integration?
RQ2: How does the school innovation climate (a school-level, or Level-2, factor) moderate the relationship between a teacher’s pedagogical leadership (an individual-level, or Level-1, factor) and their deep integration of GAI?

To account for the nested data structure—with teachers (Level 1) situated within schools (Level 2)—we utilize Hierarchical Linear Modeling (HLM). This statistical approach is specifically designed to analyze such multilevel data, allowing us to examine both individual and school-level effects simultaneously (Davidian, 2003; Haslwanter, 2016). By applying HLM to survey data from 512 teachers across 45 schools, this study aims to enhance understanding of educational technology integration. It explores a nuanced developmental pathway for teachers while providing multilevel empirical evidence on the pivotal role of school context. This research seeks to offer actionable insights for school leaders and policymakers, emphasizing the importance of fostering a supportive innovation climate to harness the transformative potential of GAI in education.

## 2 Literature review and hypothesis development

This section establishes the theoretical framework for our multilevel model. By leveraging established theories in educational technology, teacher development, and organizational innovation, we systematically formulate a series of hypotheses. Each subsection is devoted to the logical derivation of a specific hypothesis, culminating in a comprehensive theoretical model that elucidates the moderated sequential mediation process.

### 2.1 The overall effect: GAI adoption and deep pedagogical integration (H1)

The rise of GAI has led to its widespread adoption by educators, primarily due to its potential for enhancing efficiency (Kasneci et al., 2023). However, the true aim of educational technology is not mere usage but transformative pedagogical change (Ertmer and Ottenbreit-Leftwich, 2010). Deep pedagogical integration, characterized by using technology to fundamentally redesign learning tasks and create new possibilities (Hawkridge, 2011; Kohnke and Zou, 2025; Son, 2024), embodies this transformation. According to theories of innovation diffusion (Shaw et al., 2021), the initial adoption phase is crucial, introducing individuals to the innovation and setting the stage for deeper evaluation and implementation. Experiential learning theory (Kolb, 1983) asserts that direct engagement with a new tool is the essential first step for subsequent learning and development. The adoption and use of GAI offer teachers the “concrete experience” needed to explore its pedagogical benefits. This initial interaction is posited to be a direct precursor to deeper integration. To establish this foundational relationship, we propose:

*H1*: GAI adoption has a positive direct effect on its deep pedagogical integration.

### 2.2 The mediating role of teacher digital literacy (H2)

While initial adoption is crucial, its effect on deep integration is likely mediated by the development of specific competencies. Teacher digital literacy, encompassing both technical skills and the pedagogical competence to design and manage technology-enhanced learning (Fraillon et al., 2020; Santos et al., 2023), is a key intermediate outcome. This aligns with the Technological Pedagogical Content Knowledge (TPACK) framework (Ong and Annamalai, 2023), which asserts that effective technology integration requires a synthesis of technological knowledge (TK), pedagogical knowledge (PK), and content knowledge (CK). The use of GAI prompts teachers to reflect on its practical applications, limitations, and pedagogical potential, thereby enhancing their TPACK (Pan and Wang, 2025). As teachers progress from novice to proficient users, their improved digital literacy enables them to translate the tool’s features into meaningful learning activities(Yan et al., 2024b). Essentially, digital literacy acts as the bridge converting initial tool usage into sophisticated pedagogical practice. Thus, we hypothesize a simple mediation:

*H2*: Teacher digital literacy mediates the relationship between GAI adoption and deep pedagogical integration.

### 2.3 The mediating role of teacher pedagogical leadership (H3)

Pedagogical leadership, defined as a teacher’s professional agency and initiative, is a crucial mechanism. It entails the proactive effort to innovate in one’s practice and influence the wider instructional community (Reid et al., 2022). According to Self-Determination Theory (SDT), voluntarily adopting and mastering new technology like GAI fulfills the need for autonomy and competence (Ryan and Deci, 2000; Deci et al., 2017). This bolstered sense of efficacy and self-direction cultivates a leadership orientation (Shao, 2023; Wenner and Campbell, 2016). A teacher who takes the initiative to use GAI demonstrates leadership, as transformative practices demand the courage to experiment and lead change (Ghamrawi et al., 2023; Parveen et al., 2022). Thus, pedagogical leadership serves as a key mediator:

*H3*: Teacher pedagogical leadership mediates the relationship between GAI adoption and deep pedagogical integration.

### 2.4 The sequential mediation pathway (H4)

Building upon the previous hypotheses, we propose a more nuanced, developmental sequence. It is plausible that the development of digital literacy precedes the full expression of pedagogical leadership. According to theories of teacher development (Darling-Hammond et al., 2017; Rakoczy, 2022; Masoumi and Noroozi, 2023), competence often fuels confidence and agency. As teachers first adopt GAI and build their digital literacy (Path 1), they gain the necessary skills and confidence. This newfound competence then empowers them to exhibit pedagogical leadership—to experiment boldly and share their knowledge (Path 2). Finally, it is this leadership mindset that drives the ultimate conversion into deep pedagogical integration (Path 3). This “adoption→literacy→ leadership→ integration” sequence represents a logical progression of professional growth. We therefore hypothesize a chain mediation effect that provides a more complete explanation than the simple mediations proposed in H2 and H3:

*H4*: Teacher digital literacy and pedagogical leadership sequentially mediate the relationship between GAI adoption and deep pedagogical integration.

### 2.5 The moderating role of school innovation climate (H5)

The success of an individual teacher’s innovative efforts does not occur in a vacuum; it is contingent upon the organizational context (Fullan, 2016). A school’s innovation climate—the shared perception that the organization supports new ideas and risk-taking—is a critical contextual factor. Organizational theories of psychological safety suggest that a supportive climate provides the “safety net” for individuals to take risks (Edmondson and Lei, 2014; Cropley and Cropley, 2015). We argue that this climate acts as a “cross-level amplifier.” In a school with a high-innovation climate, a teacher’s pedagogical leadership is more likely to be encouraged, resourced, and ultimately translated into deep integration(Anderson et al., 2014; Bach et al., 2019). Conversely, in a restrictive climate, even a highly motivated teacher leader may find their efforts stifled (Roach and Debarbieux, 2017). The climate thus moderates the effectiveness of the final, crucial step in our proposed pathway. Therefore, we propose our final hypothesis:

*H5*: The school’s innovation climate moderates the positive relationship between teacher pedagogical leadership and deep GAI integration, such that the relationship is stronger in schools with a more supportive innovation climate.

**Figure 1:**
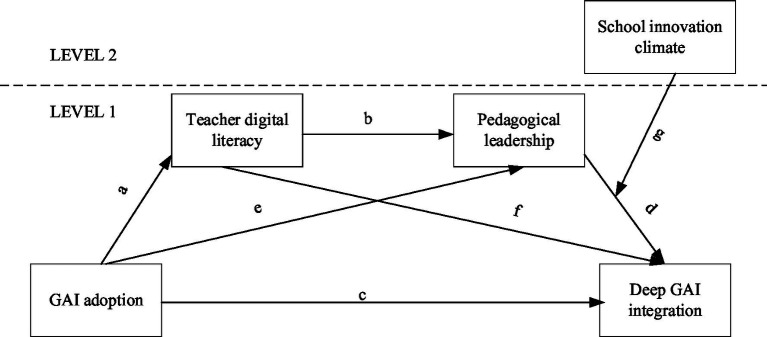
Research hypothesis model.

Based on the preceding literature review and hypothesis development, this study proposes an integrated multilevel moderated mediation model. The model, depicted in Figure 1, outlines the total effect of GAI Adoption on Deep GAI Integration (H1,path c), which is explained through three potential mechanisms: a simple mediation through Teacher Digital Literacy (H2,path a, f), a simple mediation through Teacher Pedagogical Leadership (H3,path e, d), and a more comprehensive sequential mediation pathway (H4, path a, b, d). Furthermore, the model posits that the final link in this pathway is context-dependent, with School Innovation Climate (a Level-2 variable) moderating the relationship between Teacher Pedagogical Leadership and Deep GAI Integration (H5, path g).

## 3 Methodology

This section outlines the methodological approach employed to investigate the multilevel factors influencing the deep pedagogical integration of Generative AI (GAI). It details the research design, participant sampling, measurement instruments, data collection process, and the analytical strategy used to systematically test the proposed hypotheses.

### 3.1 Research design and participants

To adequately address the research questions concerning the interplay between individual-level teacher attributes and school-level contextual factors, a cross-sectional, multilevel research design was adopted. This design is appropriate for examining relationships among variables within and between different hierarchical levels (i.e., teachers nested within schools) at a single point in time (Davidian, 2003). While the cross-sectional nature limits causal inference, it provides a robust and efficient snapshot of the complex, co-occurring mechanisms linking GAI adoption, teacher development, and organizational context, setting a critical foundation for future longitudinal work.

The target population for this study comprised K-12 teachers and their corresponding school administrators (principals or academic deans) in a major metropolitan region in Eastern China. This region was selected due to its advanced digital infrastructure and active government-led promotion of educational technology, providing a suitable context with sufficient variance in GAI adoption and integration practices.

A two-stage stratified sampling approach was utilized to secure a representative and diverse sample.

Stage 1 (School Level): 80 K-12 schools were purposefully selected from a regional database, stratified by educational level (primary, middle, high school) and funding type (public, private) to ensure heterogeneity in organizational structures and resources. From this stratified list, 50 schools were randomly invited to participate. This stratification was crucial to avoid over-representing a single type of school and to enhance the generalizability of the findings concerning school-level factors. Of these, 45 schools ultimately agreed to participate, resulting in a strong school-level response rate of 90%.

Stage 2 (Teacher Level): within each of the 45 participating schools, a list of all full-time teachers was obtained from the administration. A random subset of 15 teachers per school was then selected and invited via email to partake in an online survey. To enhance participation, two follow-up emails were dispatched over a three-week period. A total of 540 teachers responded. After excluding responses with substantial missing data on key variables (n = 28, representing 5.2% of responses), the final sample comprised 512 teachers. A Little’s MCAR test indicated that the data were missing completely at random (χ^2^(156) = 168.45, *p* = 0.24), justifying the exclusion of incomplete cases without introducing significant bias. This yielded a final teacher-level response rate of approximately 75.8% ((512/(45 schools * 15 invitations)) * 100).

The final sample of 512 teachers was nested within 45 schools, with an average of 11.4 teachers per school (ranging from 8 to 15), satisfying the recommended minimum of 8–10 units per group for multilevel modeling. Key demographic information is summarized in Table 1.

**Table 1:** Descriptive statistics of teacher and school samples.

Variable	Category/Statistic	*N*	%/Mean (SD)
Level 1: teacher characteristics (*N* = 512)			
Gender	Male	164	32.0%
	Female	348	68.0%
Age (Years)	Mean (SD)	512	35.4 (7.8)
Teaching experience (Years)	Mean (SD)	512	11.2 (6.5)
Level 2: school characteristics (*N* = 45)			
School type	Primary School	20	44.4%
	Middle School	15	33.3%
	High School	10	22.2%
Funding source	Public	35	77.8%
	Private	10	22.2%
Teachers per school	Mean (SD)	45	11.4 (2.1)

This study received ethical approval from the Institutional Review Board of Qufu Normal University (Protocol 2,025,113). All participation was strictly voluntary. Before beginning the survey, all participants (teachers and administrators) were presented with a clear informed consent form. This form detailed the study’s purpose, the estimated time for completion, the confidential and anonymous nature of their responses, and their right to withdraw at any time without penalty. To ensure confidentiality, no personally identifiable information (e.g., name, email) was collected in the final dataset. All data were stored on a password-protected, encrypted server accessible only to the primary researchers. The contribution of large language models (LLMs) was limited to assisting with language polishing and structural organization of the manuscript draft, with all final content and analysis verified by the authors.

### 3.2 Measures

All constructs were measured using scales validated in prior literature, unless otherwise specified. A rigorous translation-back-translation procedure was employed to adapt the original English-language scales into Mandarin Chinese, ensuring conceptual and semantic equivalence (Erkut, 2010). With the exception of demographic variables, all items were rated on a 5-point Likert scale (1 = *Strongly Disagree* to 5 = *Strongly Agree*). The internal consistency for each scale was assessed using Cronbach’s alpha, with all values exceeding the recommended 0.70 threshold.

#### 3.2.1 Level 1 (teacher level) variables

GAI Adoption (Independent Variable): we adapted a 4-item scale from Tondeur et al. (2015) to measure teachers’ self-reported frequency and breadth of GAI usage in their professional work. A sample item includes, “I frequently use GAI tools for my teaching-related tasks.” The scale yielded high reliability (*α* = 0.88).

Teacher digital literacy (Mediator 1): this was measured with a 6-item scale adapted from the European Framework for the Digital Competence of Educators (DigCompEdu; Santos et al., 2023). The items were selected to specifically assess pedagogical competencies related to technology, rather than purely technical skills. A sample item reads, “I am skilled at designing digital learning activities that foster students’ collaboration and critical thinking.” The internal consistency of this scale was excellent (*α* = 0.91).

Teacher pedagogical leadership (Mediator 2): a 5-item scale, based on the conceptualization of teacher leadership by Reid et al. (2022), was used to assess teachers’ perceived agency and initiative in driving instructional innovation. A sample item is, “I actively experiment with new teaching methods in my classroom, even when they are challenging.” The reliability for this scale was strong (*α* = 0.89).

Deep GAI integration (Dependent Variable): this construct was assessed using a 6-item scale developed for this study. The scale was theoretically grounded in the transformative levels (“Modification” and “Redefinition”) of the SAMR model (Kohnke and Zou, 2025; Son, 2024) to capture the extent to which GAI is leveraged to fundamentally redesign teaching tasks and create new learning opportunities. A sample item is, “I use GAI to create personalized learning paths for students that were previously impossible to implement.” The scale demonstrated excellent internal consistency (Cronbach’s α = 0.92). Prior to its use, the scale underwent expert validation with three educational technology scholars and a pilot test with 30 teachers to ensure clarity and content validity.

#### 3.2.2 Level 2 (school level) variable

School innovation climate (Moderator): this school-level construct was measured using a 7-item scale adapted from Mustafa et al. (2022). To mitigate common method bias, these data were collected from a different source. In each of the 45 participating schools, one senior administrator (e.g., principal or academic dean) completed the survey, reporting on the shared perceptions of support for innovation within the school. A sample item is, “In our school, teachers are encouraged to try out new ideas, even if they might fail.” The scale demonstrated high internal consistency (α = 0.90). Aggregation to the school level was justified by strong inter-rater agreement and within-group agreement indices calculated from a pilot phase (ICC(1) = 0.23, ICC(2) = 0.78, and rwg(j) = 0.91), confirming that perceptions were largely shared within schools and differed between them.

#### 3.2.3 Control variables

To account for potential confounding influences, we included several control variables at both levels.

Level 1: teacher age, gender (0 = male, 1 = female), and teaching experience (in years), as these factors may influence technology adoption and pedagogical practice (Hatlevik et al., 2014).

Level 2: school type (dummy coded) and funding source (0 = public, 1 = private), as these may reflect differences in resources and organizational culture.

### 3.3 Scale confirmatory factor analysis and structural validity

To ensure the robustness of our measurement model, we conducted a confirmatory factor analysis (CFA) using AMOS 26.0 to assess the structural validity of the key constructs: Generative AI Adoption, Digital Literacy, Teaching Leadership, and Deep Integration. This step is crucial for verifying that the measurement items reliably reflect their intended latent constructs and that these constructs are empirically distinct from one another, thereby establishing both convergent and discriminant validity (Hair et al., 2014).

We evaluated the model fit using a comprehensive set of established indices: the chi-square to degrees of freedom ratio (χ^2^/df), the Comparative Fit Index (CFI), the Tucker-Lewis Index (TLI), the Root Mean Square Error of Approximation (RMSEA), and the Standardized Root Mean Square Residual (SRMR). According to established guidelines, a good model fit is indicated by χ^2^/df < 3, CFI and TLI > 0.90, RMSEA < 0.08, and SRMR < 0.08 (Wolf and McNeish, 2023).

As shown in Table 2, we compared our hypothesized four-factor model against several more parsimonious, alternative models. The results demonstrate that the hypothesized four-factor model (Generative AI Adoption, Digital Literacy, Teaching Leadership, and Deep Integration as distinct factors) achieved an excellent fit to the data (χ^2^ = 245.67, df = 129, χ^2^/df = 1.90, CFI = 0.97, TLI = 0.96, RMSEA = 0.042, SRMR = 0.035). All fit indices for this model comfortably met and exceeded the recommended thresholds.

**Table 2:** Confirmatory factor analysis.

Model	χ^2^	df	χ^2^/df	CFI	TLI	RMSEA	SRMR
Hypothesized four-factor model	245.67	129	1.90	0.97	0.96	0.042	0.035
Three-factor model (DL + TL)	689.43	132	5.22	0.85	0.82	0.091	0.088
Two-factor model (DL + TL + DI)	1254.88	134	9.36	0.71	0.67	0.125	0.110
One-factor model	2103.51	135	15.58	0.52	0.46	0.168	0.154

Crucially, the fit of the hypothesized model was substantially superior to all competing models. For instance, the three-factor model, where Digital Literacy and Teaching Leadership were combined, showed a significantly poorer fit (Δχ^2^ = 443.76, *p* < 0.001). The degradation in fit was even more pronounced for the two-factor and single-factor models, indicating that the constructs in our study are not interchangeable and possess strong discriminant validity. This rigorous comparison confirms that the four latent variables are distinct and well-defined, providing a solid structural foundation for the subsequent mediation and moderation analyses.

### 3.4 Data analysis strategy

The analytical procedure was executed systematically, involving preliminary data screening, measurement model validation, and formal hypothesis testing using SPSS 26, Mplus 8.3, and HLM 8.0.

Stage 1: preliminary analysis. First, we conducted descriptive statistics and correlation analyses in SPSS. Second, to justify the use of a multilevel framework, we calculated the intraclass correlation coefficient (ICC(1)) for the dependent variable (Deep GAI Integration) by running a null (unconditional) model in HLM. The ICC(1) was 0.23, with significant between-school variance (*τ*₀₀ = 0.18, χ^2^(44) = 98.76, p < 0.001). This result indicates that 23% of the total variance in deep integration resided at the school level, confirming the necessity of employing multilevel modeling to account for the nested data structure (Davidian, 2003). Third, as detailed in Section 3.3, a confirmatory factor analysis (CFA) was performed in Mplus to validate the measurement model for the four Level-1 latent constructs.

Stage 2: hypothesis testing for mediation (H1–H4).

The mediation hypotheses were tested using multilevel structural equation modeling (MSEM) in Mplus 8.3, which can simultaneously estimate complex path models while accounting for the nested data structure. The analysis proceeded as follows:

Total Effect (H1): we first tested the total effect of GAI Adoption on Deep GAI Integration (path c) to establish a baseline relationship.

Mediation Effects (H2, H3, H4): we then specified the full moderated mediation model. The significance of the simple mediation paths (H2 and H3) and the sequential mediation path (H4) was examined using the bias-corrected bootstrap method with 5,000 resamples. This method is superior for testing indirect effects as it does not assume a normal sampling distribution. We analyzed the specific indirect effects and their 95% confidence intervals (CIs). A CI that does not contain zero indicates a statistically significant mediation effect.

Stage 3: hypothesis testing for moderated mediation (H5).

Hypothesis 5, which posits a cross-level moderation, was tested using Hierarchical Linear Modeling (HLM). This approach is ideal for modeling interactions between Level-1 and Level-2 variables.

Centering: all Level-1 predictors were group-mean centered to provide a pure estimate of the within-school effects, unconfounded by between-school differences. The Level-2 moderator (School Innovation Climate) was grand-mean centered to facilitate the interpretation of main effects and the intercept.

Model specification: the HLM model was specified as follows:


$$\text{Level}\;1\;\text{Model}:Y_{\mathrm{ij}}=\beta_{0j}+\beta_{1j}(\mathrm{TP}L_{\mathrm{ij}})+r_{\mathrm{ij}}$$

$$\text{Level}\;2\;\text{Model}:\beta_{0j}=\gamma_{00}+\gamma_{01}(\mathrm{SI}C_{j})+u_{0j}$$

$$\beta_{1j}=\gamma_{10}+\gamma_{11}(\mathrm{SI}C_{j})+u_{1j}$$

Interpretation: a statistically significant cross-level interaction coefficient (γ₁₁) would provide support for H5. If significant, we would conduct a simple slopes analysis to probe the nature of the interaction, plotting the relationship between pedagogical leadership and deep integration at high (+1 SD) and low (−1 SD) levels of school innovation climate.

## 4 Results

This section presents the statistical outcomes of the data analysis. It begins with preliminary analyses, including descriptive statistics, correlations, and validation of the measurement model. This is followed by the main findings from the multilevel modeling used to systematically test each of the proposed hypotheses (H1–H5).

### 4.1 Preliminary analyses

Descriptive statistics and Pearson correlations for all study variables are presented in Table 3. All key variables were positively and significantly correlated, providing initial support for the hypothesized relationships. Notably, Deep GAI Integration was strongly correlated with GAI Adoption (*r* = 0.58, *p* < 0.01), Teacher Digital Literacy (*r* = 0.65, *p* < 0.01), and Teacher Pedagogical Leadership (*r* = 0.69, *p* < 0.01). Furthermore, the school-level variable, Innovation Climate, also showed a significant positive correlation with Deep GAI Integration (*r* = 0.34, *p* < 0.01), suggesting that both individual and school-level factors are substantively linked to the outcome.

**Table 3:** Descriptive statistics, correlations, and validity of key study variables.

Variable	M	SD	1	2	3	4	5
Level 1 (*N* = 512)							
1. GAI Adoption	3.55	0.88	(0.85)				
2. Teacher Digital Literacy	3.68	0.91	0.62**	(0.88)			
3. Teacher Pedagogical Leadership	3.71	0.85	0.55**	0.68**	(0.86)		
4. Deep GAI Integration	3.49	0.95	0.58**	0.65**	0.69**	(0.89)	
Level 2 (*N* = 45)							
5. School Innovation Climate	3.82	0.54	0.29**	0.31**	0.35**	0.34**	(0.87)
Cronbach’s α			0.88	0.91	0.89	0.92	0.90
Composite Reliability (CR)			0.93	0.94	0.94	0.95	0.94
Average Variance Extracted (AVE)			0.72	0.76	0.75	0.80	0.76

*N* = 512 teachers in 45 schools. **p < 0.01. Diagonal values in parentheses are the square root of the Average Variance Extracted (AVE). School Innovation Climate is a Level-2 variable (*N* = 45), correlations are based on disaggregated data.

As detailed in Section 3.3, the confirmatory factor analysis confirmed the excellent structural validity of our four-factor measurement model, establishing strong convergent and discriminant validity for all latent constructs.

### 4.2 Hypothesis testing

To test the proposed hypotheses, we employed a multilevel modeling approach. The mediation hypotheses (H1–H4) were analyzed using multilevel structural equation modeling (MSEM) in Mplus 8.3. The cross-level moderation hypothesis (H5) was tested using hierarchical linear modeling (HLM) in HLM 8.0. All Level-1 predictors were group-mean centered.

#### 4.2.1 Testing the Total effect and mediation pathways (H1, H2, H3, H4)

**Figure 2:**
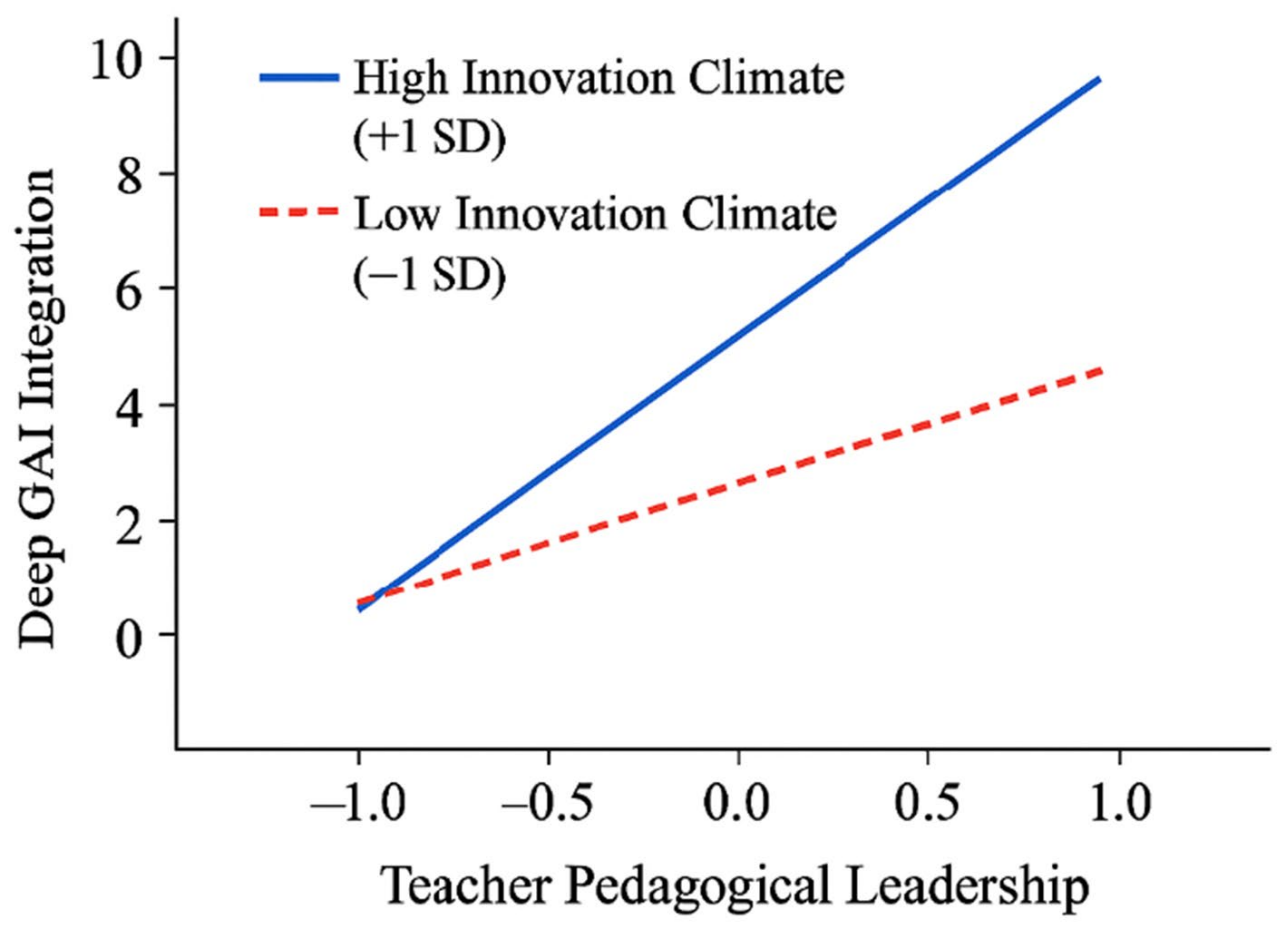
Simple slopes for the moderating effect of school innovation climate.

We first examined the overall relationship and the mediating mechanisms connecting GAI adoption to deep integration. The results of the multilevel path analysis are detailed in Table 4 and visualized in Figure 2.

**Table 4:** Results of the multilevel sequential mediation analysis.

Path	Estimate	S. E.	95% B-C CI
Direct effects			
GAI Adoption→Digital Literacy (a)	β = 0.52***	0.04	[0.44, 0.60]
GAI Adoption→Pedagogical Leadership (e)	β = 0.27***	0.05	[0.17, 0.37]
Digital Literacy→Pedagogical Leadership (b)	β = 0.45***	0.04	[0.37, 0.53]
Digital Literacy→Deep Integration (f)	β = 0.22***	0.04	[0.14, 0.30]
Pedagogical Leadership→Deep Integration (d)	β = 0.38***	0.04	[0.30, 0.46]
GAI Adoption → Deep Integration (c)	β = 0.15**	0.05	[0.05, 0.25]
Total effect			
H1. GAI Adoption → Deep Integration (c)	β = 0.41***	0.04	[0.33, 0.49]
Indirect effects			
H2. Adoption → Literacy → Integration	0.114***	0.022	[0.073, 0.160]
H3. Adoption → Leadership → Integration	0.058***	0.016	[0.029, 0.091]
H4. Adoption → Literacy → Leadership → Integration	0.089*	0.019	[0.051, 0.127]

*N* = 512. B-C CI = Bias-Corrected Confidence Interval based on 5,000 bootstrap samples. S. E. = Standard Error. *p < 0.05*,*p < 0.01, ***p < 0.001. All coefficients are standardized.

Hypothesis 1: the analysis began by establishing the total effect of GAI Adoption on Deep GAI Integration. A significant positive total effect was found (Total Effect = 0.41, *p* < 0.001), satisfying the prerequisite for mediation and providing support for H1. This indicates that, overall, greater adoption of GAI is associated with deeper levels of pedagogical integration.

Hypotheses 2, 3, and 4: we then proceeded to test the specific indirect effects using a bias-corrected bootstrap procedure with 5,000 samples. The results provided support for all three mediation hypotheses.

H2 (Mediation via Digital Literacy): the indirect effect of GAI Adoption on Deep GAI Integration through Teacher Digital Literacy was significant and positive (Indirect Effect = 0.114, 95% CI [0.073, 0.160]). This supports H2, indicating that GAI adoption enhances digital literacy, which in turn promotes deep integration.

H3 (Mediation via Pedagogical Leadership): the indirect effect through Teacher Pedagogical Leadership was also significant and positive (Indirect Effect = 0.058, 95% CI [0.029, 0.091]). This supports H3, suggesting that GAI adoption also fosters pedagogical leadership, which subsequently facilitates deep integration.

H4 (Sequential Mediation): most importantly, the full sequential mediation pathway (Adoption → Literacy → Leadership → Integration) was found to be significant and positive (Indirect Effect = 0.089, 95% CI [0.051, 0.127]). This provides robust support for H4, confirming the existence of a developmental chain where adoption builds literacy, literacy empowers leadership, and leadership drives deep integration.

After accounting for all three indirect paths, the direct effect of GAI Adoption on Deep GAI Integration remained significant but was substantially reduced (c’ = 0.15, *p* < 0.01), indicating that the relationship is partially, yet complexly, mediated by both individual and sequential pathways.

#### 4.2.2 Testing the cross-level moderating role of school innovation climate (H5)

Hypothesis 5 proposed that School Innovation Climate (Level 2) would moderate the positive relationship between Teacher Pedagogical Leadership (Level 1) and Deep GAI Integration (Level 1). The HLM results, summarized in Table 5, strongly substantiate this claim.

**Table 5:** HLM results for the moderating effect of school innovation climate.

Fixed Effects	Coefficient (*γ*)	S. E.	t-ratio	*p*-value
Model for deep GAI integration intercept, β₀ⱼ				
Intercept (γ₀₀)	3.51	0.08	43.88	<0.001
School Innovation climate (γ₀₁)	0.28	0.08	3.55	<0.01
Teaching experience (γ₀₂)	−0.09	0.04	−2.15	<0.05
School type (γ₀₃)	−0.11	0.12	−0.92	0.364
Funding source (γ₀₄)	0.05	0.13	0.38	0.703
Model for pedagogical leadership slope, β₁ⱼ				
Intercept (γ₁₀)	0.35	0.05	7.12	<0.001
School innovation climate (γ₁₁)	0.21	0.07	3.12	<0.01
Variance components	Variance	df	χ^2^	p-value
School-level variance in intercept (u₀ⱼ)	0.071	39	98.76	<0.001
Within-school variance (rᵢⱼ)	0.425			

S. E. = Standard Error. Level-1 predictors are group-mean centered. Level-2 predictors are grand-mean centered.

The model revealed significant main effects for both Teacher Pedagogical Leadership (*γ*₁₀ = 0.35, *t* = 7.12, *p* < 0.001) and School Innovation Climate (γ₀₁ = 0.28, *t* = 3.55, *p* < 0.01) on Deep GAI Integration. This indicates that, on average, stronger pedagogical leadership and a more supportive school climate are independently associated with deeper GAI integration.

Central to H5, the cross-level interaction term between School Innovation Climate and Teacher Pedagogical Leadership was positive and statistically significant (γ₁₁ = 0.21, *t* = 3.12, *p* < 0.01). This confirms a significant moderating effect. The positive coefficient indicates that the positive relationship between a teacher’s pedagogical leadership and their deep GAI integration is strengthened in schools with a more supportive innovation climate.

To probe the nature of this interaction, a simple slopes analysis was conducted, and the results are plotted in Figure 2. The analysis revealed that:

In schools with a high innovation climate (+1 SD), the positive relationship between Teacher Pedagogical Leadership and Deep GAI Integration was strong and steep (Simple Slope = 0.46, *p* < 0.001).

In schools with a low innovation climate (−1 SD), the relationship was still positive but significantly weaker and less steep (Simple Slope = 0.14, *p* < 0.05).

This pattern clearly demonstrates the amplifying role of a supportive school environment. The effectiveness of a teacher’s leadership in driving deep integration is significantly enhanced when situated within a school culture that encourages innovation.

Regarding the control variables, at the teacher level, teaching experience was found to be negatively associated with deep GAI integration (γ = −0.09, *p* < 0.05). At the school level, neither school type nor funding source emerged as significant predictors.

## 5 Discussion

This study investigated the complex mechanisms underlying teachers’ deep pedagogical integration of Generative AI. Adopting an ecological systems perspective, we tested a multilevel model that illuminates the interplay between individual professional growth and organizational context. Our findings not only validate a nuanced developmental pathway for teachers but also empirically underscore the indispensable role of a school’s innovation climate. In this section, we discuss the theoretical and practical implications of these findings, acknowledge the study’s limitations, and suggest directions for future research.

### 5.1 Theoretical implications

This study makes several significant contributions to the literature on educational technology integration and teacher development.

First, we articulate and empirically validate a specific, sequential pathway from GAI adoption to deep integration. Previous research has established links between adoption and integration (Márquez et al., 2023) but often treats the intervening process as a “black box” (Fullan, 2016). Our findings dissect this process, revealing a “competence-to-agency” developmental sequence: the significant sequential mediation effect (Indirect Effect = 0.089, *p* < 0.05) demonstrates that initial adoption enhances digital literacy, which in turn fosters the pedagogical leadership necessary for transformative practice. This sequential mediation model (Adoption → Literacy → Leadership → Integration) provides a more granular and theoretically grounded explanation than simpler models. It refines theories like TPACK (Ong and Annamalai, 2023) by illustrating a dynamic process where technological practice (adoption) builds knowledge (literacy), which then activates pedagogical agency (leadership), ultimately leading to the synthesis of all three in deep integration.

Second, by employing a multilevel framework, this study challenges the predominantly individual-centric focus of much educational technology research. While models like TAM and TPACK are invaluable, they often fall short in explaining why skilled and motivated teachers may still fail to integrate technology deeply. Our findings provide a powerful answer: the organizational context matters profoundly. The significant variance in deep integration attributable to school-level differences (ICC(1) = 23%) offers robust empirical support for the arguments of scholars like Tondeur et al. (2015), who have long advocated for moving beyond individual-level analyses. This study thus reinforces the need to view technology integration not merely as a matter of individual skill or will, but as an ecological phenomenon shaped by the systems in which teachers are embedded.

Third, and most critically, we identify the school innovation climate as a key cross-level moderator, specifying how the organizational context shapes individual efforts. Our model goes beyond merely stating that context is important; it pinpoints a specific mechanism. The significant cross-level interaction (*γ*₁₁ = 0.21, *p* < 0.01) found in our HLM analysis is a crucial contribution. It suggests that institutional culture acts as a catalyst or an inhibitor for the final, most difficult step in the integration journey. This finding extends organizational theories of innovation (e.g., Anderson et al., 2014) into the educational technology domain, demonstrating that psychological safety and institutional support for risk-taking are not just “nice-to-haves” but are essential boundary conditions for realizing the transformative potential of technologies like GAI.

### 5.2 Practical implications

The findings from this multilevel study offer actionable insights for teachers, school leaders, and policymakers committed to harnessing the transformative potential of GAI beyond superficial use. The validated sequential pathway provides a clear, evidence-based roadmap for stakeholders, emphasizing that deep integration requires a concerted effort across multiple levels of the educational ecosystem (Çelik, 2024).

For teachers, this study highlights that professional growth follows a “competence-to-agency” sequence. The path from initial adoption to deep integration necessitates a conscious effort to build profound digital literacy—understanding not just how to use GAI, but why and for what novel pedagogical purposes. To cultivate the pedagogical leadership that drives transformation, teachers can proactively form “GAI Innovation Pods,” creating localized environments of psychological safety for experimentation. Furthermore, engaging in practices such as peer coaching can translate individual exploration into shared, actionable knowledge, thereby building the collective confidence to champion wider change (Reid et al., 2022). The act of documenting and sharing transformative practices, including “intelligent failures,” is itself a crucial expression of this emerging leadership.

For school leaders, the most critical implication is the imperative to shift from being technology providers to innovation cultivators. The significant amplifying effect of a supportive school climate (*γ*₁₁ = 0.21) suggests that leadership is the fulcrum for unlocking teacher potential. Our findings provide compelling quantitative evidence for this shift: the significant cross-level interaction effect (γ₁₁ = 0.21, *p* < 0.01) demonstrates that a supportive school climate acts as a powerful amplifier for teacher-led innovation. To be specific, our simple slopes analysis revealed that the positive impact of pedagogical leadership on deep GAI integration was over three times stronger in schools with a high-innovation climate (simple slope = 0.46) compared to those with a low-innovation climate (simple slope = 0.14). This stark difference underscores that merely distributing GAI tools is insufficient; leaders must architect an “infrastructure of innovation.” This involves establishing “innovation sandboxes”—protected spaces where teachers are explicitly encouraged to test novel pedagogies without pressure for immediate success. Crucially, leaders should celebrate “intelligent failures” by publicly acknowledging thoughtful experiments that do not meet their goals, as this is a powerful mechanism for destigmatizing risk-taking and fostering genuine psychological safety (Edmondson and Lei, 2014). Such cultural leadership must be underpinned by systemic resources, including targeted professional development and collaborative planning time.

For policymakers and teacher educators, this study advocates for a paradigm shift toward a more holistic, ecological policy perspective. Our results demonstrate that deep integration is not a single event but a developmental process. The significant sequential mediation pathway (Adoption→Literacy→Leadership→Integration; Indirect Effect = 0.089) suggests that policy interventions must support teachers along this entire journey, not just at the initial adoption stage. Therefore, instead of focusing narrowly on decontextualized “how-to” workshops, professional development should be redesigned to support entire school teams, including principals, in learning about both GAI integration and the principles of leading innovative cultures (Leithwood et al., 2019). To create systemic incentives that reflect this complexity, education authorities should consider developing and integrating a “Pedagogical Innovation Index” into school evaluation frameworks. Such an index should assess not only technology adoption but also the growth of teacher literacy, the emergence of pedagogical leadership, and the perceived level of institutional support—precisely the factors our model identifies as critical. This approach would reward schools for systemic change, aligning with our core finding that context is a powerful determinant of success.

### 5.3 Limitations and future directions

While this study offers a robust multilevel framework, its limitations define a clear agenda for future research. First, its cross-sectional design provides a valuable snapshot but prevents definitive causal claims. Longitudinal studies that track teachers and schools over several years are essential to confirm the causal ordering of the “Adoption → Literacy → Leadership → Integration” chain. Second, our reliance on self-report measures, while standard, could be complemented by more objective data. Future research should triangulate findings using observational data of classroom practice, content analysis of GAI-generated lesson plans, or artifacts of student work to provide a more holistic assessment of deep integration.

First, we articulate and empirically validate a specific, sequential pathway from GAI adoption to deep integration. Previous research has established links between adoption and integration (e.g., Márquez et al., 2023) but often treats the intervening process as a “black box” (Fullan, 2016). Our findings dissect this process, revealing a “competence-to-agency” developmental sequence: the significant sequential mediation effect (Indirect Effect = 0.089, *p* < 0.001) demonstrates that initial adoption enhances digital literacy, which in turn fosters the pedagogical leadership necessary for transformative practice. This sequential mediation model (Adoption → Literacy → Leadership → Integration) provides a more granular and theoretically grounded explanation than simpler models. It refines theories like TPACK (Ong and Annamalai, 2023) by illustrating a dynamic process where technological practice (adoption) builds knowledge (literacy), which then activates pedagogical agency (leadership), ultimately leading to the synthesis of all three in deep integration(Selwyn, 2019).

Third, our measurement of School Innovation Climate relied on a single administrator’s report per school. While this provides a valuable managerial perspective, future studies could benefit from aggregating perceptions from multiple teachers to construct a more robust, shared climate variable (rwg), mitigating potential single-source bias and capturing the climate as a truly collective construct (Podsakoff et al., 2023). Finally, this study was conducted within the specific context of Eastern China, a region characterized by a more collectivist culture and strong top-down policy implementation. The powerful amplifying effect of school climate found here might itself be moderated by national cultural dimensions. Replicating this model in more individualistic Western educational contexts would be a crucial test of the cross-cultural generalizability of these ecological interactions.

Furthermore, to address the “how” and “why” behind our quantitative findings, future work would greatly benefit from incorporating a qualitative component, as suggested by the editor. For instance, conducting comparative case studies in schools with high and low innovation climates could illuminate the specific mechanisms through which a supportive environment operates—whether it is through explicit leadership behaviors, formal peer collaboration structures, or informal norms of communication. In-depth interviews with teachers who exemplify high pedagogical leadership could also provide rich, narrative accounts of their developmental journey, revealing the personal struggles, critical incidents, and subjective meanings they attach to the “competence-to-agency” pathway. Such qualitative data would not only add depth and texture to our model but also generate more nuanced, context-sensitive practical guidance for educators.

## 6 Conclusion

The integration of Generative AI in education stands at a critical juncture, where the conversation must shift from mere access to meaningful pedagogical transformation. This study demonstrates that such deep integration is not an isolated act but an emergent outcome of a dynamic interplay between individual teacher development and the broader organizational context. We have illuminated a developmental pathway where initial GAI adoption matures into genuine digital literacy, which in turn empowers teachers to assume pedagogical leadership. Yet, our most critical finding is that this individual journey is profoundly shaped by the school environment. A supportive innovation climate does not simply help; it acts as a powerful catalyst, amplifying the impact of teacher leadership and making the path to transformative practice truly viable. Therefore, to unlock the true potential of GAI, educational systems must invest as much in cultivating an “infrastructure of innovation”—built on trust, support, and shared vision—as they do in distributing innovative tools.

## Data Availability

The data analyzed in this study is subject to the following licenses/restrictions: the data presented in this study are not publicly available due to privacy and ethical concerns regarding the human participants involved. Qualified researchers may request access to the data by contacting the corresponding author. Requests to access these datasets should be directed to TD qfnudtq@qfnu.edu.cn.
